# Creating and Validating Ligase Primers to Detect Single Nucleotide Polymorphisms Associated with Atovaquone Resistance in *Plasmodium falciparum*

**DOI:** 10.4269/ajtmh.22-0700

**Published:** 2023-03-06

**Authors:** Muharib Alruwaili, Rubayet Elahi, Donelly van Schalkwyk, Colin Sutherland, Theresa Shapiro, Sean Prigge, David Sullivan

**Affiliations:** ^1^ Department of Tropical Medicine, School of Public Health and Tropical Medicine, Tulane University, New Orleans, Louisiana;; ^2^ Department of Clinical Laboratory Sciences, College of Applied Medical Sciences, Jouf University, Sakaka, Saudi Arabia;; ^3^ Department of Molecular Microbiology and Immunology, Johns Hopkins Bloomberg School of Public Health, Baltimore, Maryland;; ^4^ Department of Infection Biology, London School of Hygiene and Tropical Medicine, London, United Kingdom;; ^5^ Division of Clinical Pharmacology, Department of Medicine, Johns Hopkins University, Baltimore, Maryland

## Abstract

Atovaquone-proguanil is one of the most commonly prescribed malaria prophylactic drugs. However, sporadic mutations conferring resistance to atovaquone have been detected in recent years associated with single nucleotide polymorphisms (SNPs) in the *Plasmodium falciparum* cytochrome *b* ( *pfcytb*) gene. Monitoring polymorphisms linked with resistance is essential in assessing the prevalence of drug resistance and may help in designing strategies for malaria control. Several approaches have been used to study genetic polymorphisms associated with antimalarial drug resistance. However, they either lack high throughput capacity or are expensive in time or money. Ligase detection reaction fluorescent microsphere assay (LDR-FMA) provides a high-throughput method to detect genetic polymorphisms in *P. falciparum*. In this study, we have created primers to detect SNPs associated with clinically relevant atovaquone resistance using LDR-FMA and validated them in clinical samples. Four SNPs from *pfcytb* gene were analyzed using LDR-FMA. The results were 100% consistent with DNA sequence data, indicating that this method has potential as a tool to detect genetic polymorphisms associated with atovaquone resistance in *P. falciparum*.

## INTRODUCTION

Approximately 241 million malaria cases and 627,000 malaria-related deaths were reported globally in 2020.^1^ *Plasmodium falciparum* is the deadliest malaria parasite.^2^ If left untreated, *P. falciparum* can progress to severe disease, and death can occur within 24 hours.^2^ All non-immune travelers with a high probability of contracting malaria visiting highly endemic areas are strongly recommended to take chemoprophylactic antimalarial drugs and adhere to other preventive measures to avoid malaria complications.^3^ Atovaquone was developed in the 1990s as an anti-protozoal agent, showing activity against cytochrome b in mitochondria.^4^^,^^5^ However, because there were high rates of recrudescence, the drug has been combined with the synergistic antimalarial drug proguanil, and in 2000 the combination Atovaquone-proguanil (AP) was approved in North America and Europe for treatment and chemoprophylaxis of malaria, marketed as Malarone^®^.^4^^,^^6^^,^^7^

*Plasmodium falciparum* has demonstrated the ability to develop resistance to atovaquone.^8^ *Plasmodium falciparum* selects mutations in the cytochrome *b* (*pfcytb*) gene due to selective pressure of atovaquone, and these mutations have been reported in patients after returning from malaria-endemic countries.^4^^,^^9^ Malaria patients failing AP treatment carried parasites that exhibited point mutations at codon 268 in the *pfcytb* gene, where a single base pair change resulted in an amino acid change from tyrosine (Y) to either serine (S) or cysteine (C).^7^ Another mutation has also been reported at the same codon, where asparagine (N) substituted tyrosine (Y) from a patient who experienced a recrudescence after Malarone treatment.^10^ Some treatment failures have been reported without any mutations in the *pfcytb* gene, and these are usually attributed to poor drug absorption in some individuals.^4^^,^^6^^,^^7^

Even though parasites readily develop atovaquone resistance, it may not be as transmissible in the field as other antimalarial resistance mutations. It has been found that, although these mutations protect the parasite from atovaquone in humans, they decrease parasite fitness in the mosquito vector, limiting the ability of atovaquone-resistant parasites to spread in the field.^11^

Genetic polymorphism in the *P. falciparum* genome associated with antimalarial drug resistance has been studied using a variety of assays, such as using restriction fragment length polymorphism (RFLP).^12^^–^^14^ Although this analysis is reliable and offers an accurate assessment of previously characterized variants, it is relatively expensive, is labor intensive, and can miss rare variants.^4^^,^^14^ A relatively new approach, known as ligase detection reaction–fluorescent microsphere assay (LDR-FMA), has been used to detect a variety of resistance-associated mutations in *P. falciparum*.^14^^–^^18^ LDR-FMA offers a high-throughput system compared with other reported methods. In addition, this approach has been validated for samples stored on filter papers collected from malaria-endemic countries. Furthermore, it has been estimated that LDR-FMA is only half the cost and provides up to 10-fold improvement in throughput with similar accuracy compared with RFLP.^14^ LDR-FMA has been applied to detect single nucleotide polymorphisms (SNPs) associated with antimalarial drug resistance mutations.^14^^–^^18^ However, this approach has not been applied and tested to detect mutations associated with atovaquone resistance. Here we design and validate ligase primers that can interrogate the presence of four different, clinically relevant SNPs in the *pfcytb* gene associated with atovaquone resistance in a single-tube multiplex assay using LDR-FMA.

## MATERIALS AND METHODS

### Generation of pMA-mtDNA and pMA-mtDNAY268S/C/N plasmids.

**Figure 1.**
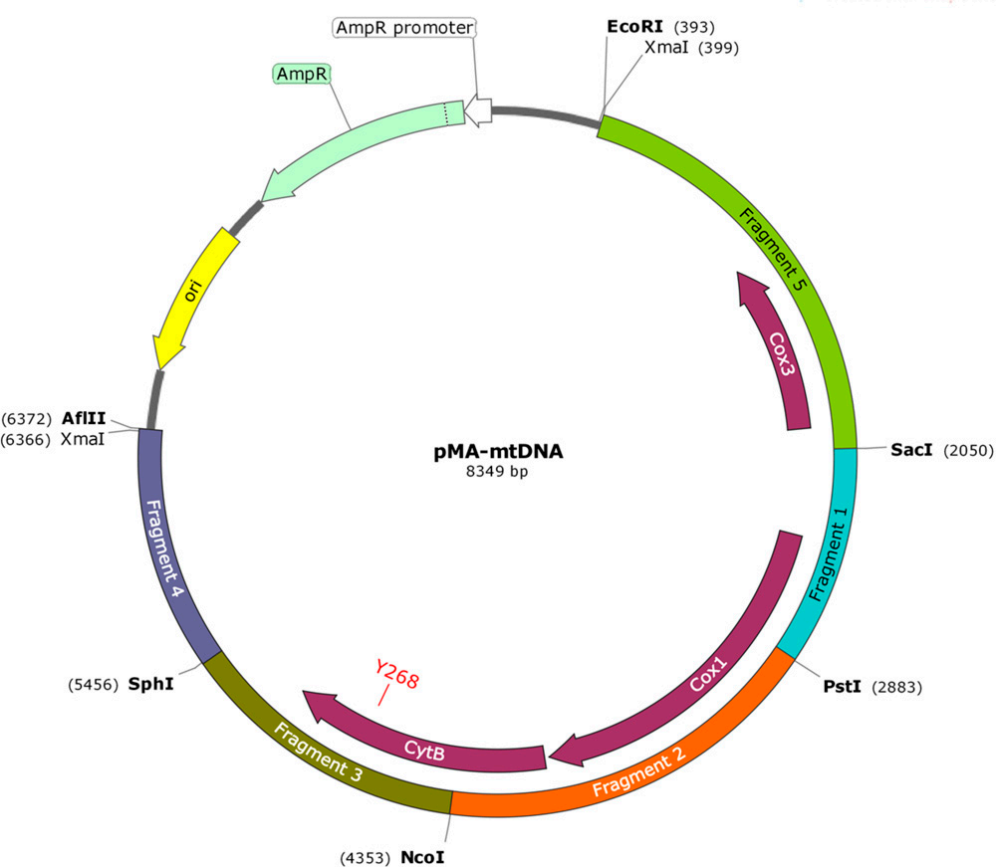
pMAmtDNA plasmid map. The plasmid contains the full-length mitochondrial genome of *Plasmodium falciparum* and relevant mutations *PfCYTB*Y268/S/C/N.

Figure 1 shows the plasmid pMA-mtDNA encoding the full-length ∼6 kb mitochondrial genome (mtDNA) of *P. falciparum*. We amplified *P. falciparum* 3D7 strain mtDNA in five fragments based on the natural existence of unique restriction enzyme sites (*Sac*I, *Pst*I, *Nco*I, *Sph*I, and *Xma*I). The primers (Supplemental Table 1) were designed so that the five polymerase chain reaction (PCR) amplicons could be pieced together to generate the full-length mtDNA. PCR fragments 1 and 2 were inserted into the *Eco*RI and *Afl*II sites of the pMA vector (GeneArt, Regensburg, Germany) to generate pMA-1 and pMA-2 plasmids, respectively. Next, we generated pMA-51 by inserting fragment 5 into the *Sac*I and *Afl*II sites of pMA-1 and generated pMA-23 by insertion of PCR fragment 3 into the *Nco*I and *Afl*II sites of pMA-2. Next, we inserted PCR fragment 4 into the *Sph*I and *Afl*II sites of pMA-23 to generate pMA-234. Finally, we digested pMA-51 with *Pst*I and *Afl*II to insert 234 from pMA-234 to generate pMA-mtDNA. The fidelity of the mtDNA sequence was confirmed by Sanger sequencing. To generate pMA-mtDNAY268S/C/N plasmids (containing mutations at position 268), we synthesized fragment 3 with the desired mutation (LifeSct LLC, Rockville, MD) and inserted it into the *Nco*I and *Sph*I sites of pMA-mtDNA.

### *Plasmodium falciparum* strains.

Genomic DNA of *P. falciparum* reference strains 3D7 and Dd2 were used in this study. The origin of these isolates has been described.^8^^,^^19^ In addition, four plasmid DNAs carrying *P. falciparum* cytochrome *b* gene with *pfcytb* Y268, *pfcytb* 268S, *pfcytb* 268C, and *pfcytb* 268N were used as positive controls.

### Clinical samples.

Deidentified clinical samples used in this study were from patients who presented to The Johns Hopkins Hospital (IRB: 00009164). Additional clinical samples were obtained from Walter Reed Army Institute of Research. Clinical isolate HL1701 was collected from a patient who failed Malarone treatment in the United Kingdom and adapted to long-term in vitro culture.

### *pfcytb* gene amplification.

Polymerase chain reaction was conducted in a 25-µL reaction mixture containing 160 nM forward and reverse primer (Supplemental Table 2), 200 nM deoxynucleotide triphosphates, 1.25 units of *Taq* DNA polymerase, and 2 µL of DNA in 1X *Taq* buffer. The reaction was performed in the following thermocycling conditions: 95 °C for 4 minutes; 30 cycles of 95 °C for 30 seconds, 56 °C for 45 seconds, 72 °C for 30 seconds; and final extension at 72 °C for 4 minutes. PCR products were validated using 2% gel electrophoresis, and the expected amplicon size was confirmed.

### Ligase detection primer design.

**Figure 2.**
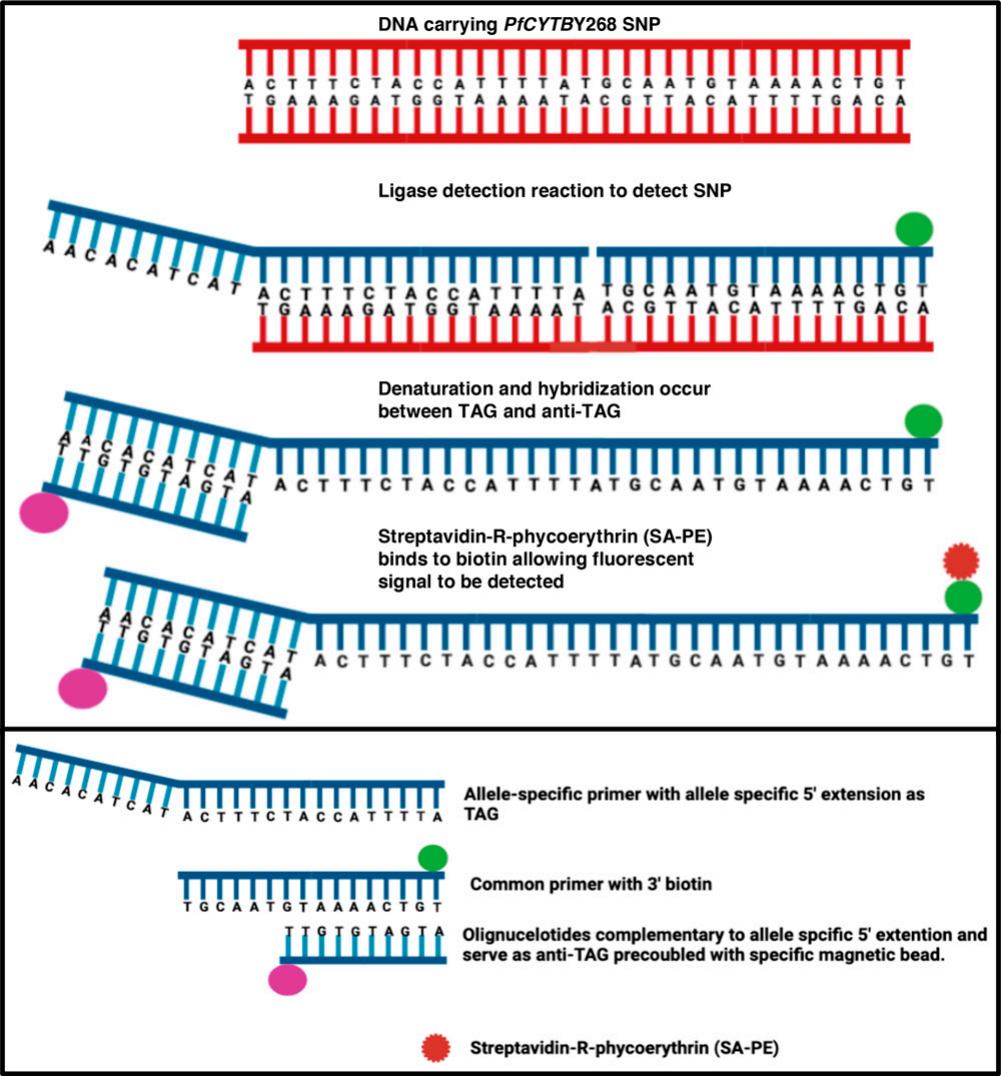
An overview of ligase detection reaction fluorescent microsphere assay steps. The forward and reverse primers in Supplemental Table 2 generate a 784 amplicon from genomic DNA (red nucleotides) containing the region of interest. The blue allele–specific primers bind 20 base pairs upstream of the Y268 position and the common primer (blue with green biotin) is 20 base pairs downstream. The ligase joins the two blue oligonucleotides that form the bead TAG-allele specific primer–common primer–biotin, which is detected. SNP = single nucleotide polymorphism.

We designed four allele-specific primers and one common primer to target four different SNPs at codon 268 in the *pfcytb* gene. These four SNPs represent one wild-type (*pfcytb* Y268) and three mutant SNPs (*pfcytb* 268S, *pfcytb* 268C, and *pfcytb* 268N) that have been associated with atovaquone resistance. All the three mutations have been identified from patients after treatment failures with atovaquone.^7^^,^^10^ Each allele-specific primer was synthesized with a 24-nucleotide extension at the 5′ end called the TAG sequences. The allele-specific primers bind to a specific sequence found upstream of the SNPs. We also designed a common primer with biotin attached to the 3′ end. The common primer binds to the conserved sequence downstream of the SNPs, which is conserved in all sequences of interest. Anti-TAGs are 24 nucleotides pre-coupled with specific magnetic beads that are complementary to 24 nucleotide extension in allele-specific primers (TAGs). Figure 2 provides an overview of the steps of the LDR-FMA procedure. The sequence of allele-specific primers and the common primer and the anti-TAG sequences can be found in Supplemental Tables 3 and 4, respectively. Magnetic beads coupled with anti-TAGs are commercially available from Luminex Corporation (Austin, TX).

### Ligase detection reaction.

Ligase detection reaction containing allele-specific and common primers was used to interrogate four possible SNPs at codon 268 in the *pfcytb* gene. PCR products of *pfcytb* were used as DNA templates. Ligase detection reactions were carried out in a 15-µL reaction mixture containing 10 nM allele-specific and common primers for each SNP, two units of *Taq* DNA ligase, and 2 µL PCR products in 1X *Taq* ligase buffer. The reaction was performed using thermocycler conditions (95 °C for 1 minute followed by 32 cycles of 95 °C for 15 seconds and 58 °C for 2 minutes) as previously described.^14^

### Hybridization with magnetic beads.

The products of LDR were hybridized with specific magnetic beads; 1,000 beads were added corresponding to each allele-specific primer. The magnetic bead hybridization was performed using 1.5× tetramethylammonium chloride (TMAC) buffer containing 3 M TMAC, 50 mM Tris-HCl (pH 8), 3 mM ethylenediaminetetraacetic acid, and 0.1% *N*-lauroylsarcosine sodium salt. Hybridization was conducted with LDR products (5 µL) added to 60 µL of the TMAC–bead mixture with the following thermocycler conditions: 95 °C for 3 minutes and 37 °C for 45 minutes. Hybridized products were stained by adding 6 µL of a mixture containing 1:50 of streptavidin-R-phycoerythrin and 0.1% bovine serum albumin in 1.5× TMAC buffer and then incubated for 15 minutes at 37 °C as previously described.^14^ The results were read as a median fluorescence intensity (MFI) using MAGPIX instrument with xPonenet software (Luminex).

### Sanger sequencing.

All clinical samples used in this study were sequenced to confirm the genotype and compared with LDR-FMA. For sequencing, an 1,848-bp fragment (containing the 1,131 bp *pfcytb* open reading frame) was amplified with primers (Supplemental Table 5) and Phusion DNA polymerase (New England Biolabs, Ipswich, MA) from lysates of clinical isolates. The lysates were prepared by incubating 10 µL of whole blood diluted in 200 µL phosphate-buffered saline at 90 °C for 5 minutes. The PCR products were verified as a single band of predicted size by gel electrophoresis before sequencing. Sequences were aligned using NCBI Blast with *P. falciparum* 3D7 cytochrome *b* (PF3D7_MIT02300).

## RESULTS

Initially we investigated the ability of LDR-FMA to precisely detect SNPs in *pfcytb*. To do this, we used *P. falciparum* 3D7 and Dd2 strain DNA carrying wild-type *pfcytb* Y268 and four different plasmids carrying wild-type (*pfcytb* Y268) or mutation (*pfcytb* 268S, *pfcytb* 268C, *pfcytb* 268N) DNA. Our analysis showed that LDR-FMA was able to detect all SNPs precisely showing MFI more than 30-fold higher than background (Table 1). The results of LDR-FMA were 100% concordant with known genotypes.

**Table 1:** MFI readings for LDR-FMA of two reference strains of *Plasmodium falciparum* (3D7 and Dd2) and four plasmids harboring wild-type *pfcytb* or SNPs at codon 268 associated with atovaquone resistance

Strain/plasmid	MFI (arbitrary units), *pfcytb* gene			
	Y268	268S	268C	268N
3D7	2,882*	75	83	75
Dd2	**2,607**	78	88	80
*pfcytb* Y268	**3,945**	85	99	85
*pfcytb* 268S	97	**3,696**	98	94
*pfcytb* 268C	89	88	**3,076**	83
*pfcytb* 268N	86	85	92	**3,354**
*pfcytb* Y268/S/C/N 1:1:1:1	**1,044**	**509**	**1,976**	132

LDR-FMA = ligase detection reaction fluorescent microsphere assay; MFI = median fluorescence intensity; *pfcytb* =* Plasmodium falciparum* cytochrome *b*; SNPs = single nucleotide polymorphisms.

* Values in bold type indicate positive allele signals.

**Figure 3.**
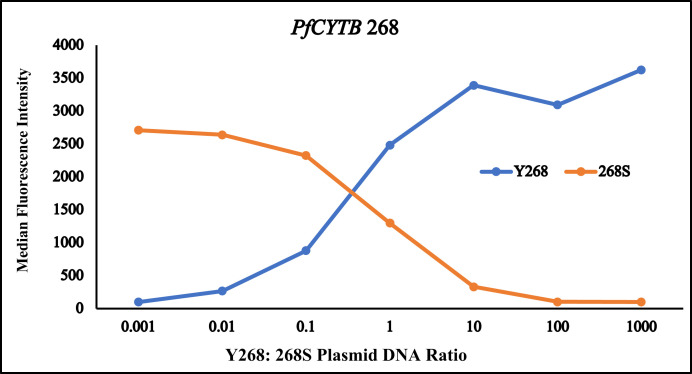
DNA from two plasmid DNA carrying either *PfCYTB* Y268 or *PfCYTB* 268S single nucleotide polymorphisms (SNPs) were mixed at different ratios. Identification of each SNP was assessed using ligase detection reaction fluorescent microsphere assay.

Next, we tested the ability of LDR-FMA to detect mixed isolates because polyclonal infections are very common in many malaria-endemic areas. We mixed the Y268 plasmid DNA with 268S plasmid DNA at ratios of 1:1, 10:1, 100:1, and 1,000:1. We also mixed 268S DNA with Y268 DNA at the same ratios. In both Y268/268S and 268S/Y268 mixed populations, we were able to clearly identify minor SNPs representing 10% of the total population (Figure 3). Interestingly, the minor Y268 allele was detected from Y268/268S mix at a 1:100 ratio, but the minor 268S allele was not detected from 268S/Y268 at a 1:100 ratio (Figure 3). This finding implied that detection sensitivity might not be equal between these SNPs. In fact, when we mixed DNA of four plasmids Y268, 268S, 268C, and 268N at a 1:1:1:1 ratio, the 268C mutant produced the highest MFI signal, followed by Y268 (Table 1). In both of these cases, the MFI signals were 10- to 20-fold higher than the background, whereas 268S showed only 5-fold higher MFI signal than the background. For the 268N mutant, the MFI signal was similar to background. These results suggest that there might be primer and or amplicon competition between SNPs and detection of one SNP may influence the detection of other.

After establishing that our LDR-FMA method can precisely detect and differentiate SNPs at position 268 of the *pfcytb* gene, we tested 14 clinical samples to determine the suitability of this method for detecting mutations. All these samples were from malaria patients treated with atovaquone. We were able to detect one 268S mutant in the United Kingdom sample (HL1701), whereas the rest were Y268 wild type. In all of the clinical isolates, the MFI was at least 10-fold higher than the background (Table 2). To confirm our data, we sequenced the *pfcytb* gene of 10 out of 14 clinical isolates, and our LDR-FMA data were 100% consistent with the sequencing results. In addition, we were able to show that our newly designed LDR primers are specific to *pfcytb* 268 positions in *P. falciparum*. To verify the specificity of the newly designed LDR primers, PCR and LDR-FMA were performed using DNA from a *P. malariae*–infected patient. We did not observe any amplified product following PCR, suggesting no cross-reactivity of our primers. As expected, the MFI of patient M from LDR-FMA was similar to background (Table 2).

**Table 2:** MFI reading for LDR-FMA of 14 clinical samples

Sample*	MFI (arbitrary units), *pfcytb *gene			
	Y268	268S	268C	268N
1	3,121	85	103	92
2	2,269	73	84	77
3	1,729	81	91	80
4	1,378	75	79	75
5	2,345	70	78	69
6	1,776	77	86	80
7	1,027	85	95	88
8	1,550	82	85	81
9	3,355	102	164	102
10	3,376	84	119	88
11	3,579	88	110	95
12	**1,429** †	90	180	90
HL1701	119	**3,002**	335	116
M‡	112	91	95	84
Negative control¶	128	87	95	87

LDR-FMA = ligase detection reaction fluorescent microsphere assay; MFI = median fluorescence intensity; *pfcytb* =* Plasmodium falciparum* cytochrome *b*.

* Clinical samples 1–8 were from Walter Reed Army Institute of Research; clinical samples 9–12 and M were from The Johns Hopkins Hospital. HL1701 is a clinical isolate from a patient who failed Malarone treatment.

† Readings in bold type indicate positive allele signals.

‡ Clinical sample from patient infected with *Plasmodium malariae*.

¶ No DNA template was added.

## DISCUSSION

In this study, we have applied LDR-FMA methodology to detect SNPs associated with atovaquone resistance in *P. falciparum*. For the first time, we were able to show the results of LDR-FMA using parasite DNA of laboratory-adapted *P. falciparum* as well as plasmid DNA carrying the *pfcytb* gene containing all SNPs of interest. We demonstrated that all SNPs were identified using our LDR primers. In addition, we demonstrated that LDR-FMA can be used to detect atovaquone conferring SNPs at *pfcytb* 268 position from clinical isolates. Our data on the clinical isolates were 100% consistent with the genotypes of clinical isolates confirmed by Sanger sequencing. Furthermore, LDR-FMA with our LDR primers were species specific; the assay only detects SNPs in the cytochrome *b* gene of *P. falciparum.* The primer binding region of cytochrome *b* genes of *P. falciparum* and *P. malariae* have sufficient mismatches to result in this species specificity (Supplemental Figure 1).

Our study has some limitations, First, our clinical isolate series included a single example of a resistant variant (HL1701-268S genotype), and therefore the 268N and 268C genotypes were not represented. Second, the LDR-FMA approach is designed to detect established gene variants but would be unable to identify newly emerging resistant genotypes. However, the ease of multi-plexing remains a major advantage over the RFLP approach.

AP is not only one of the most prescribed prophylactic antimalarial drugs, but it is also considered the drug of choice for treatment of established infection in many non–malaria-endemic areas. However, resistance to atovaquone has emerged, and treatment failures following atovaquone have been reported in patients who experienced recrudescence. Three variants at codon 268 (*pfcytb* 268S, *pfcytb* 268C, and *pfcytb* 268N) of the *pfcytb* gene in *P. falciparum* have been proposed as molecular markers for atovaquone resistance.^7^ Although these variants have good predictive value for AP treatment failure due to recrudescence, patients with poor drug absorption may present with poor clearance dynamics in the absence of any *pfcytb* mutation.^4^ Nevertheless, these molecular markers provide insight into *P. falciparum* evolution and parasite response to atovaquone selective pressure, and monitoring molecular markers associated with resistance may provide valuable information regarding the development and spread of resistant parasite strains in the field should these variants become established in natural parasite populations. LDR-FMA offers an efficient genotyping method with a high throughput capability. We have shown robust results that support the usefulness of this approach to detect SNPs associated with atovaquone resistance.

## CONCLUSION

The newly designed LDR primers could be used to monitor SNPs associated with atovaquone resistance in the *pfcytb* gene. We showed that this approach can detect and differentiate *pfcytb* 268S mutations in clinical samples from the wild-type *pfcytb* Y268. LDR-FMA is an efficient genotyping method with high-throughput capacity advantages over other reported systems, such as RFLP.

## Supplemental files


Supplemental materials
