# Supplementary material for: Creating and Validating Ligase Primers to Detect Single Nucleotide Polymorphisms Associated with Atovaquone Resistance in Plasmodium falciparum
Source: Am J Trop Med Hyg. 2023 Mar 6;108(4):777–82. doi: 10.4269/ajtmh.22-0700 (PMC10077009; doi:10.4269/ajtmh.22-0700)
Supplement: Supplementary file 1 [file tpmd220700.SD1.pdf]

Supplementary materials:

Table 1: Primers used for generating and sequencing pMA-mtDNA plasmids. Restriction enzyme sites are underlined, overlapping sequencing with the pMA vector in italics.

**Primer**	**Sequence (5’ → 3’)**	**Purpose**
**SacI.InF.F**	*GATAACAATTGAATTC* GGTTCGCCGGGGATAACAGG	Forward for fragment 1
**PstI.InF.R**	*TGAGGCCCAGCTTAAG* GTCCATCCAGTTCCACCACC	Reverse for fragment 1
**PstI.InF.F**	*GATAACAATTGAATTC* CTGCAGCAGAATTTGGTGGTGG	Forward for fragment 2
**NcoI.InF.R**	*TGAGGCCCAGCTTAAG* CCCCATGGTAAGACATAACCAACG	Reverse for fragment 2
**NcoI.InF.F**	*GATAACAATTGAATTC* CGTTGGTTATGTCTTACCATGGGG	Forward for fragment 3
**SphI.InF.R**	*TGAGGCCCAGCTTAAG* GCATGCAATACCGAACATTTATCG	Reverse for fragment 3
**SphI.InF.F**	*GATAACAATTGAATTC* GTTCGGTATTGCATGCCTGGTG	Forward for fragment 4
**XmaI.InF.R**	*TGAGGCCCAGCTTAAG* CCCGGGAAACCGGCGCTTCC	Reverse for fragment 4
**XmaI.InF.F**	*GATAACAATTGAATTC* CCCGGGTATCCAATCCAGTGC	Forward for fragment 5
**SacI.InF.R**	*TGAGGCCCAGCTTAAG* GAGACGACATGGAGGTGCC	Reverse for fragment 5
**mito1.seq.F**	CCGCTGTCGCTGGGACTGTATGG	Sequencing
**mito2.seq.F**	GGTAATGCTGCCATTGATGTAGCATTAC	Sequencing
**mito3.seq.F**	GGAATTATACCTTTATCACATCCTGATAATGC	Sequencing
**mito4.seq.F**	CTACTTCCAGCAGCCATTTTTGGTTCAG	Sequencing
**mito5.seq.F**	GGAAACACACTTCCCTTCTCGCC	Sequencing
**mito6.seq.F**	GATGTTAATGCAGGATATGAAACTAGATGTGCTTTTATATTTG	Sequencing
**mito1.seq.R**	CTTTACGTTAAGGGCGTAAAATTACCTTTCCGGC	Sequencing
**mito2.seq.R**	CCCAATCAAATTGGATGGTGTTGGCTGG	Sequencing
**pMA.seq.F**	CGACGGCCAGTGAGCGCG	Sequencing
**pMA.seq.R**	GACCATGTTAATGCAGCTGGCACG	Sequencing

Table 2: Forward and reverse primer sequences and amplicon size used to amplify *PfCYTB* gene of *P. falciparum*.

***PfCYTB* primer**	**Sequence** (5' → 3')	**Amplicon size**
**Forward primer**	GGAATTATACCTTTATCACATCCTGATAATGC	784 base pair
**Reverse primer**	GCATGCAATACCGAACATTTATCG	

Table 3: Ligase Detection Reaction Primers for *P. falciparum PfCYTb* gene.

***PfCytb* codon (268)**	**Sequence** (5' → 3')	**Bead ID**
**Y268**	ttcaattcaaatcaaacacatcatGAATGGTACTTTCTACCATTTTA	**64**
**268S**	tcttactaatttcaatactcttacGAATGGTACTTTCTACCATTTTC	**66**
**268C**	ctttatcaaattctaattctcaacGAATGGTACTTTCTACCATTTTG	**73**
**268N**	cataaatcttctcattctaacaaaTGAATGGTACTTTCTACCATTTAA	**75**
**Common primer**	/5Phos/ TGCAATGTTAAAAACTGTTCCAAG /3Bio	

Table 4: Anti-TAG ID and sequences complementary to 24 nucleotides (TAGs) bound to each allele-specific primer.

**Anti-TAG ID**	**Sequence** (5' → 3')	**Bead ID**	**Allele**
**Anti-TAG 64**	ATGATGTGTTTGATTTGAATTGAA	64	Y268
**Anti-TAG 66**	GTAAGAGTATTGAAATTAGTAAGA	66	268S
**Anti-TAG 73**	GTTGAGAATTAGAATTTGATAAAG	73	268C
**Anti-TAG 75**	TTTGTTAGAATGAGAAGATTTATG	75	268N

Table 5: Sequencing primers of *pfcytb* used in sanger sequence.

Primer name	Sequence (5' → 3')
CytBSeq F	GGTAATGCTGCCATTGATGTAGCATTAC
CytBSeq F1	CGTTGGTTATGTCTTACCATGGGG
CytBSeq F2	GGAATTATACCTTTATCACATCCTGATAATGC
CytBSeq R	GCATGCAATACCGAACATTTATCG

A
P. falciparum GGAATTATACCTTTATCACATCCTGATAATGC 737
P. malariae GGTATTATACCATTATCTCATCCAGATAATGC 510

B
P. falciparum CG-ATAAATGTTCGGTATTGCATGC 1489
P. malariae AGTATAAATGTTCGGTATTGCATGC 1263

C
P. falciparum GAATGGTACTTTCTACCATTTTATGCAATGTTAAAAACTGTTCCAA 827
P. malariae GAATGGTATTTTTTACCATTTTATGCAATGTTAAAAACAATACCTA 600

Figure 1: A: DNA sequence alignment of *P. malariae* and *P. falciparum CYTB* gene of forward primer target region. B: DNA sequence alignment of *P. malariae* and *P. falciparum CYTB* gene of reverse primer target region. C: DNA sequence alignment of *P. malariae* and *P. falciparum CYTB* gene of LDR primers target region. Differences between aligned sequences are in bold and highlighted by underline**.**
